# TGF-β1-mediated transition of resident fibroblasts to cancer-associated fibroblasts promotes cancer metastasis in gastrointestinal stromal tumor

**DOI:** 10.1038/s41389-021-00302-5

**Published:** 2021-02-06

**Authors:** Hyunho Yoon, Chih-Min Tang, Sudeep Banerjee, Antonio L. Delgado, Mayra Yebra, Jacob Davis, Jason K. Sicklick

**Affiliations:** 1 Department of Surgery, Division of Surgical Oncology, University of California, San Diego, CA USA; 2 Moores Cancer Center, University of California, San Diego, CA USA; 3 Department of Surgery, University of California, Los Angeles, CA USA

**Keywords:** Sarcoma, Cell migration

## Abstract

Cancer-associated fibroblasts (CAFs) are the most abundant cells in the tumor microenvironment. Crosstalk between tumor cells and CAFs contributes to tumor survival in most epithelial cancers. Recently, utilizing gastrointestinal stromal tumor (GIST) as a model for sarcomas, we identified paracrine networks by which CAFs promote tumor progression and metastasis. However, the mechanisms by which CAFs arise in sarcomas remain unclear. Here, RNA sequencing analysis revealed that transforming growth factor-β1 (TGF-β1) is highly expressed in both tumor cells and CAFs. To determine the functional role of TGF-β1, we treated normal gastric fibroblasts (GFs) with recombinant TGF-β1, which caused the GFs to adopt a more stellate morphology, as well as increased the mRNA expression of CAF-mediated genes (*CCL2, RAB3B*, and *TNC*) and genes encoding fibroblast growth factors (*FGF*s). Moreover, while either GIST or CAF conditioned media enhanced the transition from GFs to CAFs, a TGF-β1-blocking antibody attenuated this effect. Transwell migration assays revealed that the TGF-β1-mediated transition from GFs to CAFs enhanced tumor cell migration. This migratory effect was abrogated by an anti-TGF-β1 antibody, suggesting that TGF-β1 secreted from GIST cells or CAFs is associated with GIST migration via GF-to-CAF transition. In addition, the murine spleen-to-liver metastasis model showed that GF pre-treated with TGF-β1 promoted GIST metastasis. Collectively, these findings reveal unappreciated crosstalk among tumor cells, CAFs, and normal resident fibroblasts in the stroma of sarcomas, which enhances a GF-to-CAF transition associated with tumor migration and metastasis.

## Introduction

A gastrointestinal stromal tumor (GIST), the most common sarcoma, is most often driven by oncogenic *KIT* or *PDGFRA* mutations^1–3^. As a result, anti-KIT/PDGFRA tyrosine kinase inhibitors (TKIs) are commonly used to treat these tumors. However, 50% of metastatic GISTs treated with imatinib, the first-line treatment, will develop drug-resistance within 20 months of starting therapy [median progression-free survival (PFS) 20.4 month]^4^. The objective response rates for later line anti-GIST TKIs, sunitinib, regorafenib, and ripretinib are only 7.0%, 4.5%, and 9.4%, respectively^5–7^. Due to the high rates of recurrence, alternative therapeutic targets are needed to effectively treat GIST.

Cancer-associated fibroblasts (CAFs) are the most abundant cells in the tumor microenvironment (TME). It is well established that crosstalk between epithelial cancers and CAFs can promote tumor progression and chemoresistance^8–10^. Specifically, CAFs can promote a favorable biological environment for tumor growth by secreting cytokines, chemokines, and growth factors. Over time, the spatial and temporal interactions of tumor cells and stromal cells create a symbiotic relationship in which the tumor receives a growth advantage from the CAFs, and vice versa^11^. In addition, our previous study showed paracrine fibroblastic support in GIST promotes tumor growth and metastasis^12^. These studies suggest that CAFs might be an alternative target for treating both epithelial cancers and sarcomas.

CAFs originate from various cell types, including resident fibroblasts, epithelial cells, and bone marrow-derived mesenchymal stem cells. Cytokines, chemokines, and growth factors can enhance their transition into CAFs in tumor stroma^13–15^. Transforming growth factor-β1 (TGF-β1), a ligand of the TGF-β receptor complex, plays important roles in many biological processes by activating TGF-β signaling, which promotes tumorigenesis and metastasis^16–18^. Moreover, normal fibroblasts derived from bone marrow-derived mesenchymal stem cells in pancreatic cancer stroma have been reported to transition into CAFs in response to TGF-β signaling, which enhances tumor growth and invasion^13^. However, little is known regarding the role of TGF-β signaling in the stroma of sarcomas.

Here, we present a detailed mechanism for the transition of gastric fibroblasts (GFs) into CAFs in GIST, and how this transition promotes tumor migration and metastasis. First, TGF-β1 secretion from GISTs and CAFs enhances a transition from normal resident fibroblast to CAFs. In turn, these ligand-dependent events lead to increased tumor motility. Overall, this study suggests that the TGF-β1-mediated GF-to-CAF transition in the TME plays an important role in sarcoma migration and metastasis, implying that this paracrine network might be a potential target for treating sarcomas.

## Results

### CAFs and GIST cells induce a GF-to-CAF transition

**Fig. 1:**
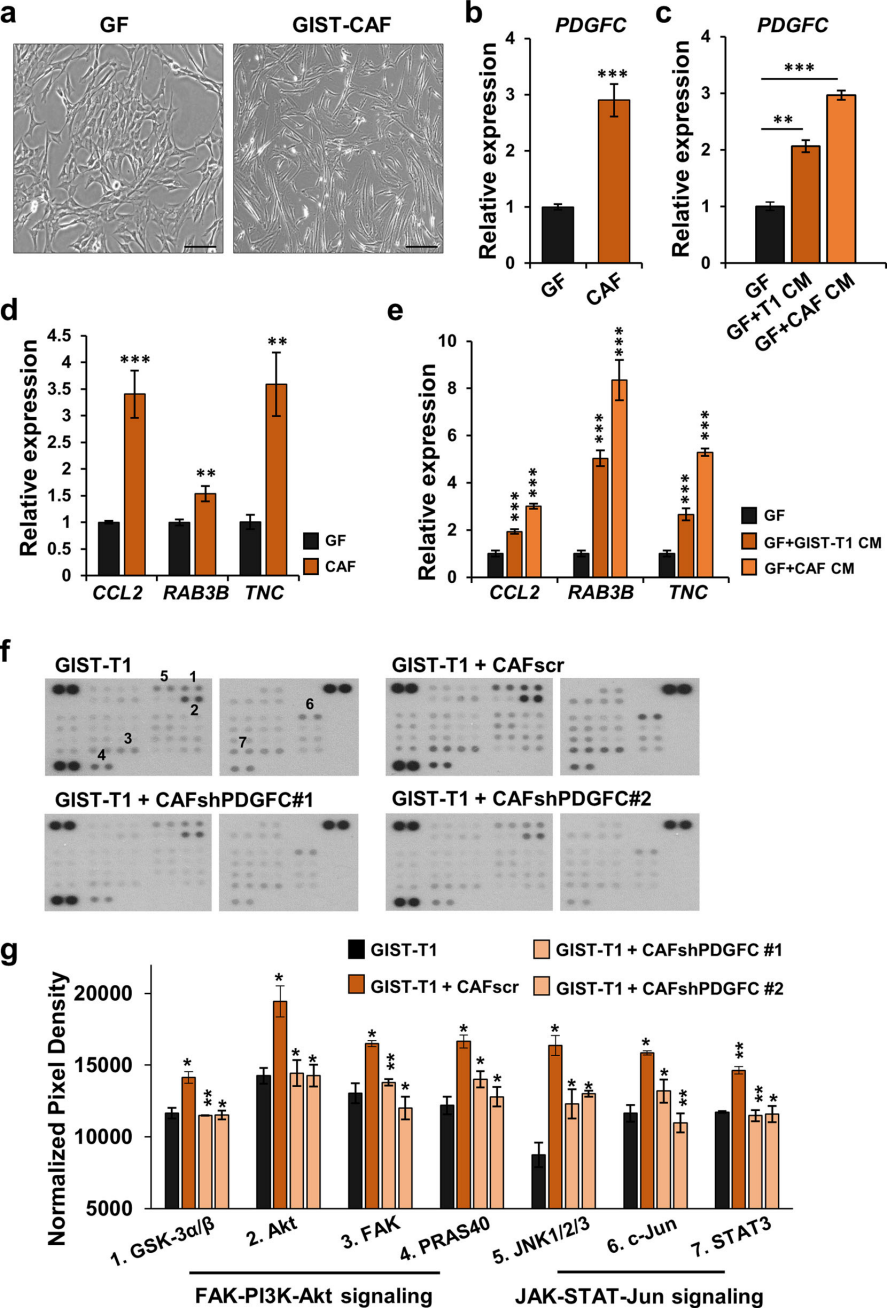
GIST cells and CAFs enhance a GF-to-CAF transition. **a** The images of gastric fibroblasts (GFs) and GIST-CAFs. Scale bars, 50 µm. **b** Expression of *PDGFC* mRNA in GFs and CAFs by quantitative RT-PCR (qPCR). **c** The effects of GIST-T1 conditioned media (CM) and CAF CM treatment on *PDGFC* expression in GFs. After GFs were treated with CM of GIST-T1 and CAFs for 48 h, the expression level of *PDGFC* was determined by qPCR. **d** Expression of CAF markers (*CCL2*, *RAB3B*, and *TNC*) in GFs and CAFs by qPCR. **e** The effects of GIST-T1 CM and CAF CM treatment on CAF markers expression in GFs. All graphs show mean ± SEM, and *p* values were represented by Student’s *t* test or ANOVA analysis. **p < 0.01, ***p < 0.001. **f** Phosphokinase array in GIST-T1 treated with CAFscr CM, CAFshPDGFC #1 CM, or CAFshPDGFC #2 CM. **g** The average signal (pixel density) was normalized with positive control and analyzed among samples. All graphs show mean ± SEM, and *p* values were represented by ANOVA analysis. *p < 0.05; **p < 0.01.

Recently, we discovered that there are CAFs in GIST (Supplementary Fig. S1a), and platelet-derived growth factor C (PDGFC) secreted from CAFs promotes tumor progression and metastasis via a paracrine PDGFC-PDGFRA-SLUG axis^12^. Since the CAFs were isolated from gastric GIST, we obtained a commercially available normal GF line to compare with CAFs. To understand the differences between resident GFs and CAFs isolated from human GIST, we compared the morphology and mRNA expression of *PDGFC*, as a functional biomarker of CAFs, in these cells. We observed that CAFs had a more stellate morphology and higher expression of *PDGFC* as compared to GFs (Fig. 1a, b). In addition, CAFs from GISTs with different mutations (i.e., *KIT* or *PDGFRA*) and distinct locations (i.e., stomach and rectum) were isolated and characterized. The quantitative polymerase chain reaction (qPCR) analysis showed that *PDGFC* mRNA was highly expressed in all CAF lines (Supplementary Fig. S1b).

We then cultured the GFs with either GIST-T1 or CAF conditioned media (CM). Both CM increased mRNA expression of *PDGFC* in GFs (Fig. 1c). In addition, we compared mRNA expression of CAF-mediated genes (*CCL2*, *RAB3B*, and *TNC*) in GFs and CAFs. *CCL2*, *RAB3B*, and *TNC* have known CAF markers in gene set enrichment analyses (GSEA; MISHRA_CAF_UP)^19^. The expression levels of *CCL2*, *RAB3B*, and *TNC* were also increased in CAFs compared to GFs (Fig. 1d; Supplementary Fig. S1c). CM from GIST-T1 cells and CAFs resulted in increased mRNA expression of *CCL2*, *RAB3B*, and *TNC* in GFs (Fig. 1e), suggesting that CM of GIST and CAFs can promote a GF-to-CAF transition.

To further investigate the functional role of PDGFC secreted from CAFs in GIST, a phosphokinase array was probed to compare CM from CAFscramble (scr), CAFshPDGFC #1, and CAFshPDGFC #2 in GIST-T1. Notably, CAFscr CM significantly activated several phosphokinase proteins, including JNK1/2/3, AKT, FAK, PRAS40, c-Jun, and STAT3, while these effects were abrogated by loss of PDGFC (Fig. 1f, g). In addition, as shown in supplementary Fig. S1d, CAFs markedly induced phosphorylation of PDGFRA and PDGFRB, while this effect was decreased by the loss of PDGFC. Because PDGFC is not known to activate PDGFR-β/β, these results suggested that CAF-mediated PDGFC secretion affects both PDGFR-α/α homo-dimerization and PDGFR-α/β hetero-dimerization, supporting our previous finding that CAF-mediated PDGFC secretion enhances GIST progression and metastasis. Together, these data demonstrated that PDGFC could be a CAF marker that modulates an aggressive phenotype of GIST cells.

### FGFs are overexpressed in CAFs

**Fig. 2:**
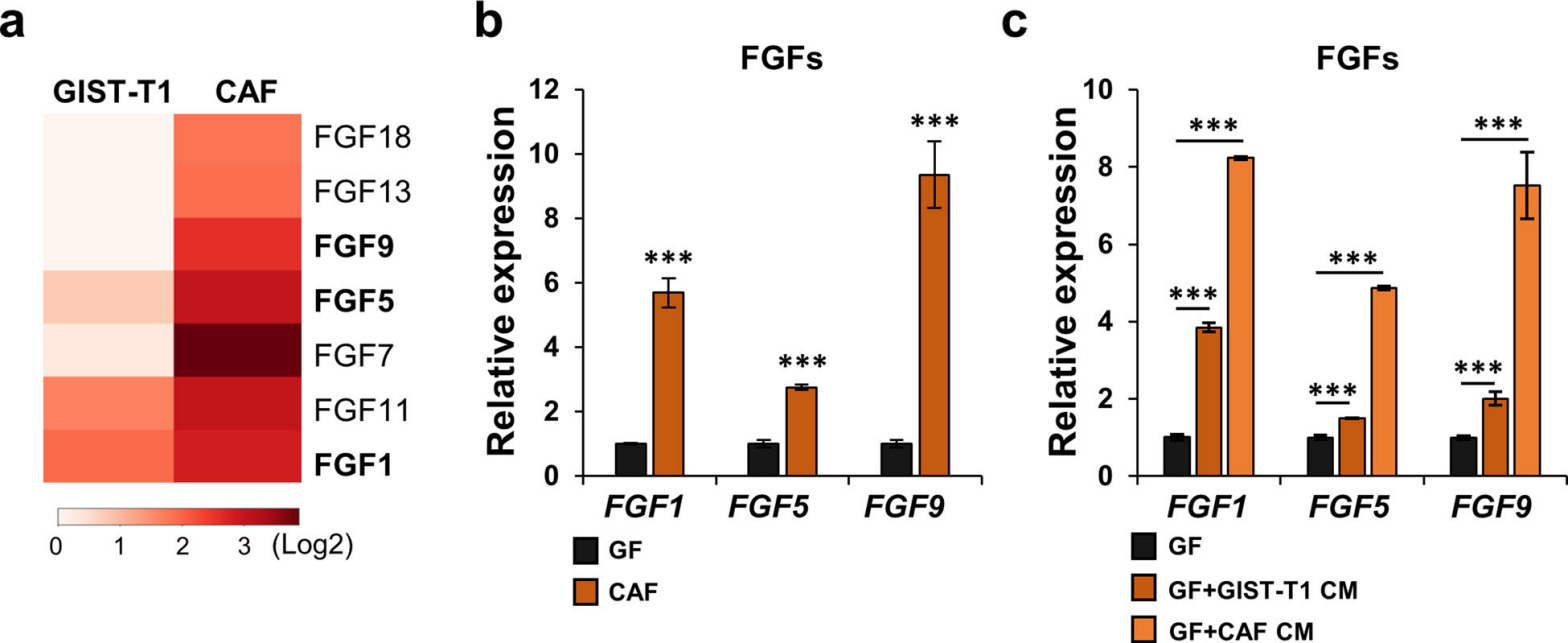
*FGFs* are overexpressed in CAFs. **a** Heatmap of the expression levels of *FGF* genes in GIST-T1 and CAFs analyzed from RNA-seq (GSE143547). **b** Expression of *FGF1*, *FGF5*, and *FGF9* in GFs and CAFs by qPCR. **c** GIST-T1 CM and CAF CM enhanced the expression of *FGF1*, *FGF5*, and *FGF9* in GF. The level of expression was evaluated by qPCR. All graphs show mean ± SEM, and *p* values were represented by Student’s *t* test or ANOVA analysis. **p < 0.01, ***p < 0.001.

Next, we performed RNA sequencing analyses (RNA-seq) using GIST-T1 cells and CAFs to characterize the CAFs (GSE143547). Interestingly, RNA-seq revealed that genes encoding various fibroblast growth factors (FGFs) were overexpressed in CAFs (Fig. 2a). These genes are known to be upregulated in many cancers, including prostate, breast, and colon cancer, as well as are associated with poor survival by promoting tumor growth, metastasis, and angiogenesis^20–22^. Thus, we next evaluated the expression of these *FGF* genes by qPCR. Among them, CAFs expressed more *FGF1*, *FGF5*, and *FGF9* expression than GFs (Fig. 2b). In addition, these FGF-encoding genes were significantly increased in GFs with the application of CM from either GIST-T1 cells or CAFs (Fig. 2c), indicating that the expression of these genes might influence the GIST phenotype.

### TGF-β1 is highly expressed in GISTs and CAFs

**Fig. 3:**
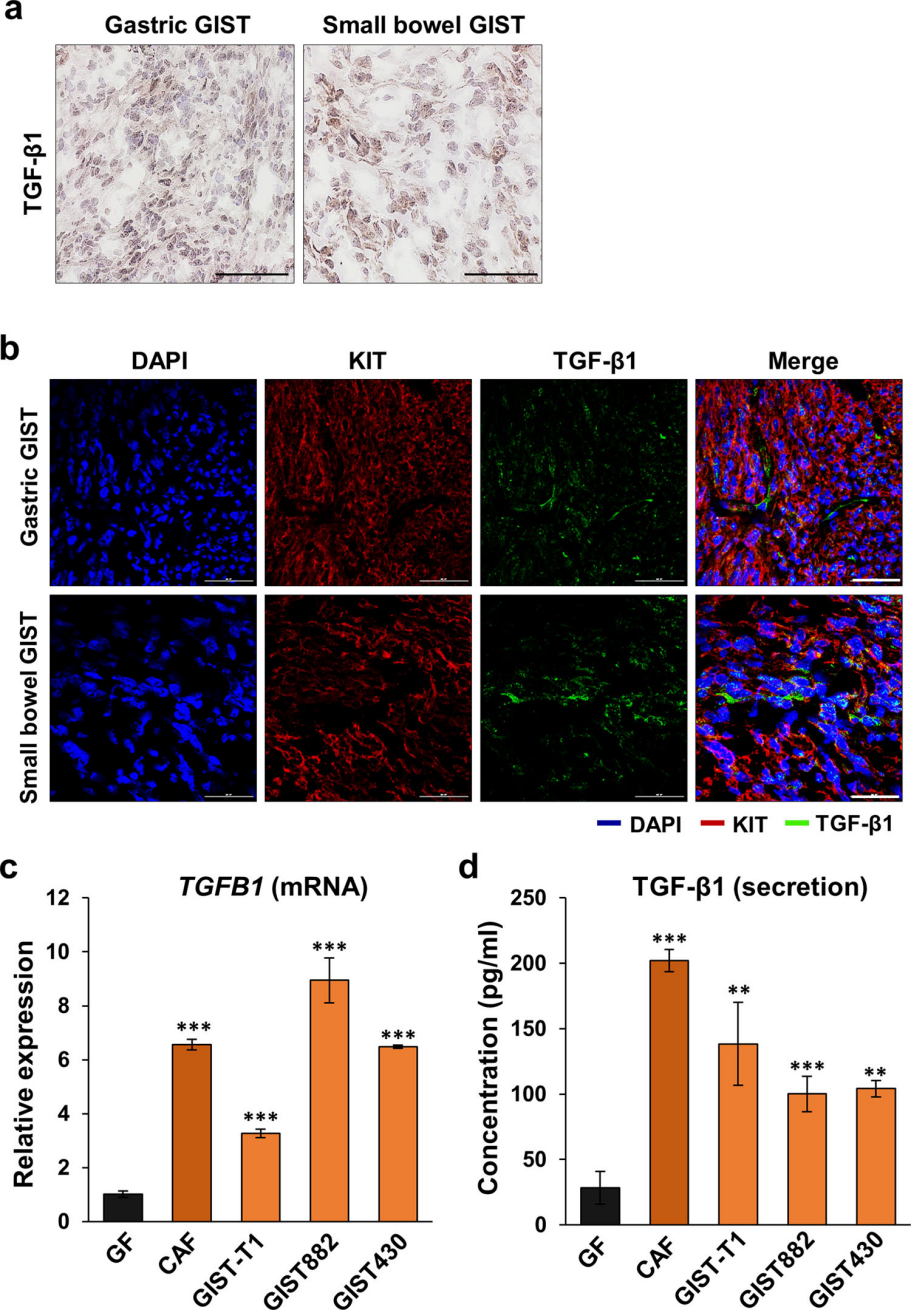
TGF-β1 is highly expressed in GIST cells and CAFs. **a** Representative immunohistochemistry (IHC) images with staining for TGF-β1 in the frozen tumor sections collected from human GISTs harboring mutant *KIT*. Scale bars, 100 µm. **b** Representative immunofluorescence (IF) images of TGF-β1 (green), KIT (GIST marker; red), and DAPI (nuclei; blue) staining in the resected sections. Scale bars, 50 µm. **c** mRNA expression of *TGFB1* in GFs, GIST-T1, GIST882, GIST430, and CAFs by qPCR. **d** Comparison of TGF-β1 levels measured by enzyme-linked immunosorbent assay (ELISA) in GFs, GIST-T1, GIST882, GIST430, and CAFs. After each cell line was seeded for 48 h in the absence of FBS, the supernatant was collected to perform ELISA. All graphs show mean ± SEM, and *p* values were represented by ANOVA analysis. **p < 0.01, ***p < 0.001.

TGF-β signaling contributes to various biological processes that promote tumorigenesis and metastasis^16,17^. In pancreatic cancer stroma, for example, normal fibroblasts convert into CAFs via TGF-β signaling, and this process is associated with tumor growth and invasion^13^. Our RNA-seq data also revealed that *TGFB1* was expressed in both GIST-T1 (transcripts per kilobase million; TPM, 724) and CAFs (TPM, 4548). Given this observation, we performed immunohistochemical (IHC) and immunofluorescent (IF) staining using human GIST frozen sections with an anti-TGF-β1 antibody. Both staining procedures demonstrated that TGF-β1 was highly expressed in resected human GIST (Fig. 3a, b; Supplementary Fig. S2a, b). Next, to determine the levels of *TGFB1* mRNA and TGF-β1 secretion, we performed qPCR and ELISA in GFs, CAFs, and GISTs. These data showed that both mRNA and protein levels of TGF-β1 were overexpressed in GIST cultures and CAFs compared to GFs (Fig. 3c, d). In addition, to test whether TKI treatment influences the secretion of TGF-β1, we performed qPCR of *TGFB1* in GIST-T1 treated with imatinib and sunitinib. Neither imatinib nor sunitinib treatment influenced *TGFB1 mRNA* expression (Supplementary Fig. S2c), suggesting that tyrosine kinase-mediated signaling in GIST cells did not affect TGF-β1 expression.

### TGF-β1 promotes a GF-to-CAF transition, which increases GIST migration

**Fig. 4:**
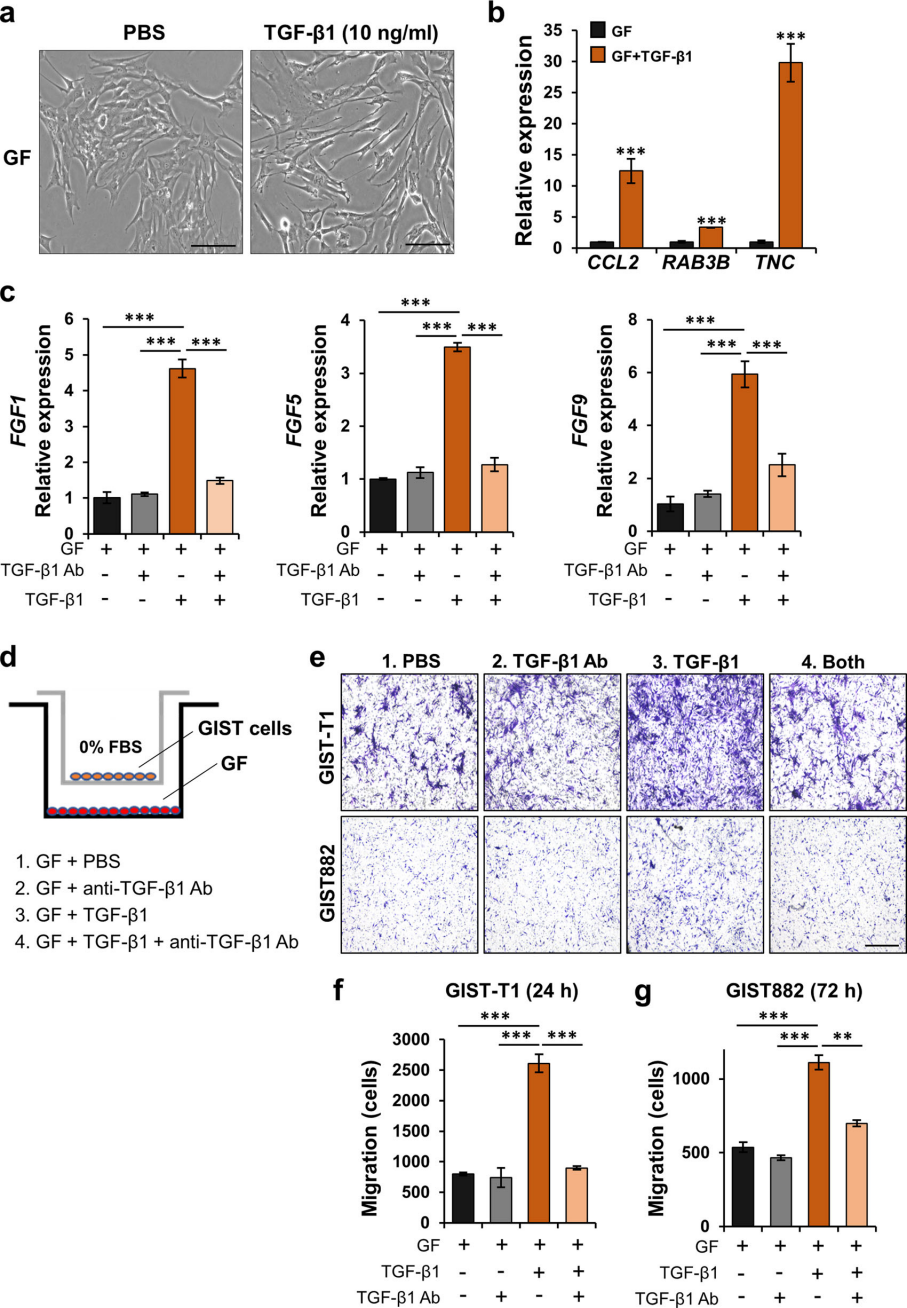
TGF-β1 is associated with a GF-to-CAF transition, which increases GIST migration. **a** Photographic images of GFs treated with human recombinant TGF-β1. Scale bars, 50 µm. GFs were treated with TGF-β1 (10 ng/mL) for 48 h. **b** Effect of TGF-β1 treatment on mRNA expression of CAF markers (*CCL2*, *RAB3B*, and *TNC*) in GFs by qPCR. **c** Effect of anti-TGF-β1 blocking antibody on the expression of *FGF1*, *FGF5*, and *FGF9* in GFs treated with TGF-β1. GFs were treated with TGF-β1 (10 ng/mL) and/or anti-TGF-β1 blocking antibody (1 µg/mL) for 48 h. The expression levels of these genes were measured by qPCR. **d** Experimental design for Transwell migration assay of anti-TGF-β1 blocking antibody. After GFs were seeded on the bottom, TGF-β1 (10 ng/mL) and anti-TGF-β1 blocking antibody (1 µg/mL) were added for 24 h. **e**–**g** Representative images (**e**) of the migrated GIST cells stained with 0.05% crystal violet in the indicated group and quantitative data in GIST-T1 cells (**f**) and GIST882 cells (**g**) analyzed by ImageJ software. Scale bars, 200 µm. All graphs show mean ± SEM, and *p* values were represented by Student’s *t* test or ANOVA analysis. **p < 0.01, ***p < 0.001.

We next explored whether TGF-β1 is sufficient for the transition of resident GFs to CAFs. Given that both GIST-T1 and CAFs overexpressed TGF-β1, we investigated the effects of this growth factor on GFs. Human recombinant TGF-β1 (10 ng/mL) treatment of GFs was associated with the development of a more stellate morphology and increased mRNA expression of *CCL2*, *RAB3B*, and *TNC* (Fig. 4a, b). TGF-β1 treatment also increased mRNA expression of *PDGFC*, *FGF1*, *FGF5*, and *FGF9*. However, the elevated expression of these TGF-β1-mediated *FGF* genes and *PDGFC* was abrogated by an anti-TGF-β1 blocking antibody (Fig. 4c; Supplementary Fig. S3a, b). Interestingly, GFs stimulated by TGF-β1 significantly increased mRNA expression of *TGFB1* in GFs (Supplementary Fig. S3c), supporting the notion of autocrine and paracrine signaling, leading to transdifferentiation from GFs to CAFs. Moreover, Transwell migration assays showed that GFs treated with human recombinant TGF-β1 (10 ng/mL) strongly enhanced migration of GIST-T1 and GIST882 cells, while this effect was significantly decreased by an anti-TGF-β1 blocking antibody (Fig. 4d–g). To test whether an isotype control for the TGF-β1 blocking antibody influences GIST migration, we performed Transwell migration assays with an isotype control antibody (mouse IgG) in GIST cells. This did not affect tumor cell migration (Supplementary Fig. S3d). Together, these data indicated that TGF-β1 is involved in GF-to-CAF transition, which leads to increased GIST migration.

### TGF-β1 secretion from GIST cells or CAFs enhances cell migration

**Fig. 5:**
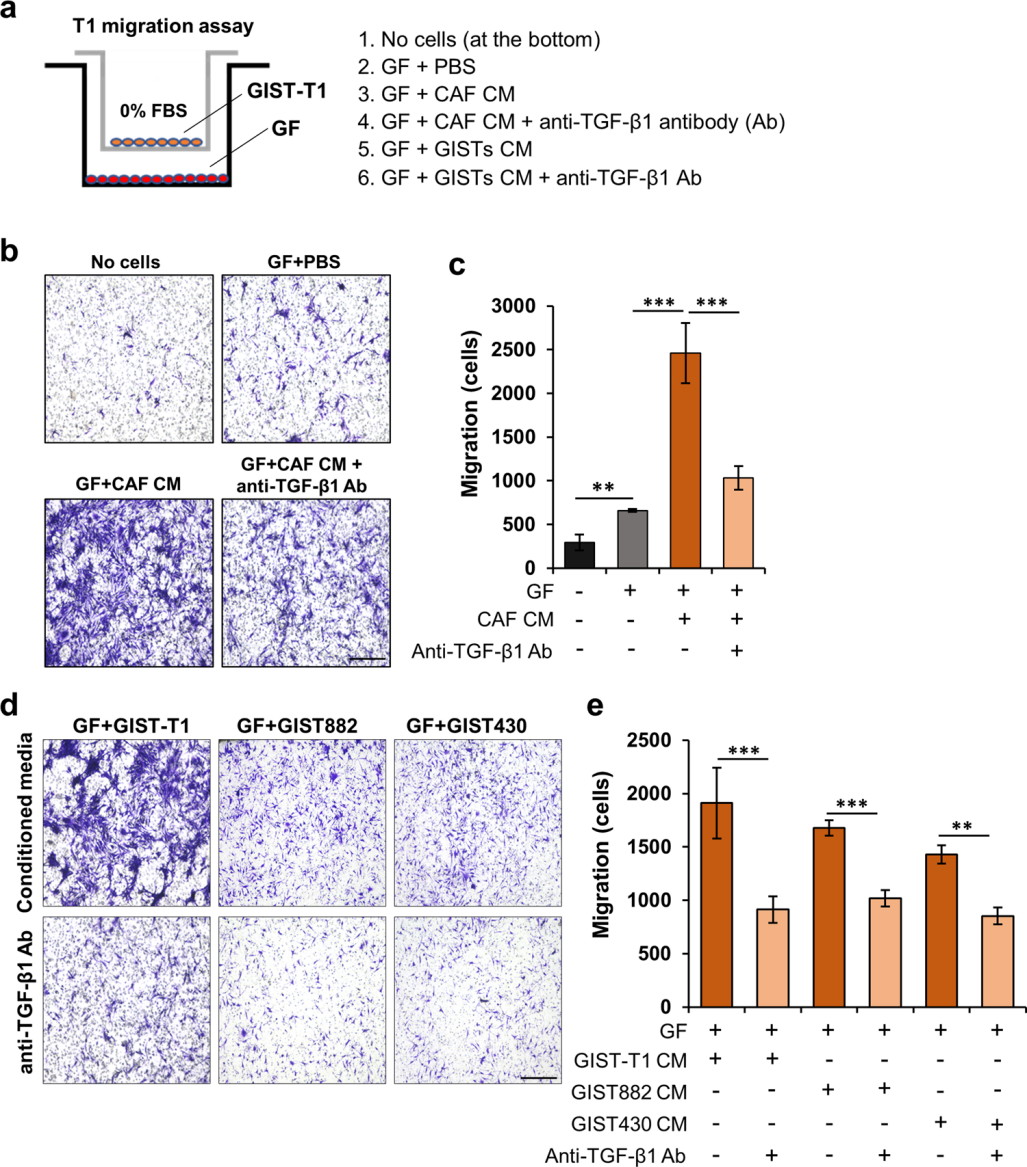
TGF-β1 secreted from GIST-T1 cells and CAFs is involved in increased GIST migration. **a** Experimental design of the Transwell migration assay. **b**, **c** Representative images (**b**) and quantitative data (**c**). GFs were treated with PBS (control), CAF CM, and/or TGF-β1 blocking antibody (1 µg/mL) for 24 h in the bottom well. The migrated GIST-T1 cells were imaged using a BZ-X800 Life Science Microscope and analyzed by ImageJ software. **d** Representative images showing Transwell migration assay of TGF-β1 blocking antibody in GFs treated with CM of GIST-T1, GIST882, and GIST430. **e** Quantitative data of the migrated GIST-T1 cells in the indicated conditions. Scale bars, 200 µm. Graphs show mean ± SEM, and *p* values were represented by ANOVA analysis. **p < 0.01, ***p < 0.001.

To investigate the effect of TGF-β1 secreted from GIST cells or CAFs, we performed Transwell migration assays with CAF CM, GIST CM, and anti-TGF-β1 blocking antibody (Fig. 5a). The migration assays showed that GFs with CM from CAFs strongly enhanced GIST-T1 cell migration compared to GF only, while the effect was markedly decreased by anti-TGF-β1 blocking antibody (Fig. 5b, c). Moreover, given that TGF-β1 was highly expressed in GIST lines, we performed Transwell migration assays with CM of GIST cell lines. The assays showed that CM of GISTs (GIST-T1, GIST882, and GIST430) in GFs increased GIST-T1 migration, while this effect was abrogated by anti-TGF-β1 antibody (Fig. 5d, e). These data suggested that TGF-β1 secreted from CAFs and various GIST lines with different KIT mutations can enhance a resident GF-to-CAF transition, which leads to increased GIST cell migration.

### The TGF-β1-mediated transition of GF to CAF promotes GIST metastasis in vivo

**Fig. 6:**
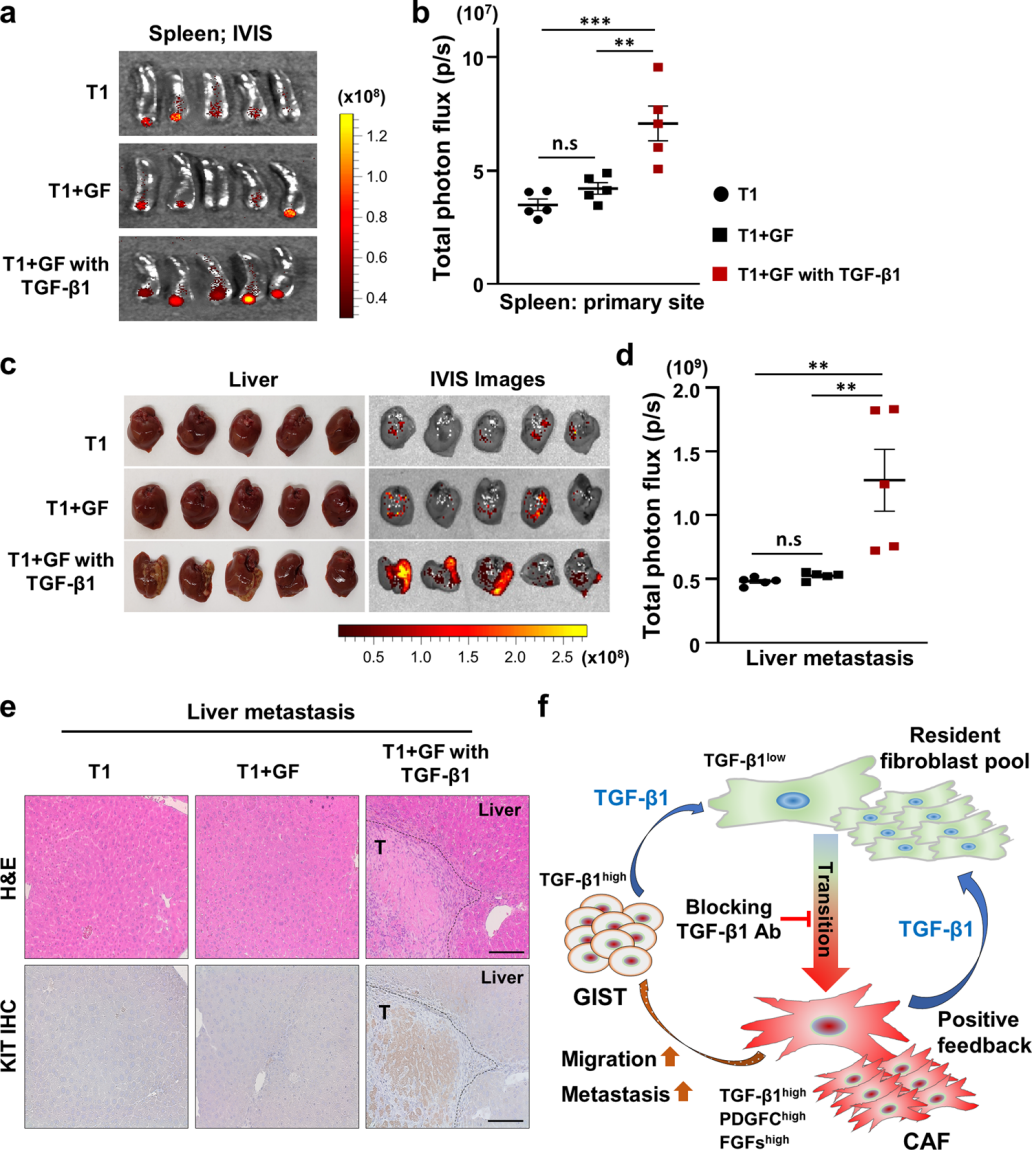
TGF-β1-mediated transition of GF to CAF promotes GIST metastasis. **a**, **b** Effects of a transition from GF to CAF on the murine spleen-to-liver metastasis model. The mice were injected with GFP-labeled T1, T1 + GF, and T1 + GF pre-treated with TGF-β1. After 3 weeks, all mice were sacrificed. The harvested spleens and livers from each mouse were analyzed using the IVIS imaging system. IVIS images (**a**) of the spleen and quantification (**b**) analyzed from total photon flux (p/s) of the spleen. *p* Values were represented by ANOVA analysis. **p < 0.01, ***p < 0.001. n.s. nonsignificant. **c** Photographic images (left) and IVIS images (right) of the liver in the indicated group. **d** Quantitative data were analyzed by total photon flux (p/s) on the metastatic liver. *p* Values were represented by ANOVA analysis. **p < 0.01. **e** Representative H&E images (top) and IHC (bottom) stained with anti-KIT antibody in the tumor sections collected from livers. Scale bars, 100 µm. **f** Proposed model of TGF-β1-mediated transition of GFs to CAFs in the GIST stroma. TGF-β1 secretion from CAFs and GIST cells enhances a GF-to-CAF transition, which promotes GIST migration via paracrine signaling networks.

We next examined the metastatic effect of TGF-β1-mediated transition from GF to CAF using our murine spleen-to-liver metastasis model. The spleens of nude mice (*n* = 5 per group) were injected with a green fluorescent protein (GFP)-labeled GIST-T1, GFP-labeled T1 + GFs, and GFP-labeled T1 + GFs pretreated with TGF-β1. The mixture of GIST-T1 cells with GFs pre-treated with TGF-β1 increased tumor formation at the injection site and liver metastases (Fig. 6a–d; Supplementary Fig. S4a, b). H&E staining and IHC with anti-KIT antibody using formalin-fixed paraffin-embedded (FFPE) liver sections from each group also supported that TGF-β1-mediated GF to CAF transdifferentiation increased GIST liver metastases (Fig. 6e; Supplementary Fig. S4c). IHC analysis in the same sections did not reveal FSP-1-positive cells in the liver, suggesting that only GIST cells stimulated (i.e., preconditioned) by CAFs (or stimulated GFs) metastasized to the liver (data are now shown). Together, these data demonstrated that TGF-β1 secretion from CAFs and GISTs enhances a GF-to-CAF transition, which promotes GIST migration and metastasis via paracrine signaling (Fig. 6f).

## Discussion

Cancer cells are in constant communication with various cells in the tumor stroma. CAFs are one of the major components in the TME, and crosstalk between cancer cells and CAFs contributes to tumor growth, invasion, and metastasis, as well as drug resistance^23–25^. In our previous study, we showed for the first time that CAFs isolated from resected human GIST play important roles in GIST progression and metastasis, suggesting the concept that targeting CAFs may represent a novel therapeutic target for treating sarcomas.

CAFs have been studied extensively and the processes of CAF development are generally known in many carcinomas. For instance, hypoxia-inducible factor-1α (HIF-1α) and CXCL12 induced by oxidative stress have been shown to enhance the transition of resident fibroblasts to CAFs in breast cancers^26^. In melanoma, normal endothelial cells can differentiate into activated fibroblasts via TGF-β1-induced endothelial-to-mesenchymal transition^27^, which then promotes cancer progression. These studies suggested that CAFs can originate from various cell types through chemokines, cytokines, and growth factors secreted from tumor stroma. In contrast, very little is known about CAF origins in sarcomas. But, given the critical role of CAFs in GIST as a sarcoma model, we next addressed how these CAFs are derived in sarcomas. Evidence for the mechanism of CAF development in the sarcoma biology was obtained from our RNA-seq data that revealed overexpression of *TGFB1* in both GIST cells and CAFs. Using resected GIST tissues, we confirmed high levels of TGF-β1 expression by IHC and IF staining. TGF-β1 is the most well-known isoform of the TGF-β subfamily, and TGF-β1 expression contributes to the development of various diseases, including cancers^28–30^. Given these observations, we hypothesized that TGF-β1 secreted from tumor cells and CAFs is a key mediator of the transition of normal resident GFs to CAFs. Indeed, we observed that TGF-β1 influenced the morphology and gene expression of GFs. Specifically, treatment with GIST and CAF CM caused resident GFs to adopt a more stellate morphology, and induced the expression of CAF-mediated genes *CCL2*, *RAB3B*, and *TNC* (GSEA; MISHRA_CAF_UP)^19^, while anti-TGF-β1 blocking antibody treatment inhibited this effect, suggesting that TGF-β1 secreted from tumor cells and CAFs plays an important role for a transition from resident fibroblasts to CAFs.

In addition, we found that GFs stimulated by TGF-β1 significantly increased the mRNA expression of *TGFB1* in GFs. Given the observation, we performed a Tanswell migration assay with TGF-β1 neutralizing antibody in the stimulated GFs since TGF-β1 is well-known as a key regulator of tumor cell migration through induction of epithelial–mesenchymal transition (EMT) in various cancers^31^. The data showed that TGF-β1 neutralizing antibody in the stimulated GFs significantly decreased GIST cell migration as compared to isotype control antibody treatment (Supplementary Fig. S4d, e). These data suggested that TGF-β1 plays an important role for GIST cell migration, as well as for GF to CAF transdifferentiation.

Our previous and present studies have shown that PDGFC could be a putative biomarker for CAFs in GIST. Elevated expression of PDGFC in CAFs promoted tumor growth and metastasis via the enhanced SLUG-mediated EMT^12^. Here, we provided additional evidence that the expression of FGF-encoding genes can be another putative marker of CAFs in sarcoma biology. FGF secretion is strongly associated with tumor progression and metastasis in many tumors^20–22,32^. The activation of GFs by TGF-β1 led to high expression levels of several genes, including *FGF1*, *FGF5*, and *FGF9*. Those *FGF*-encoding genes may play an unappreciated role in GIST.

Herein, we demonstrated that the GFs activated by TGF-β1 promoted GIST cell migration and metastasis in vitro and in vivo, suggesting that GIST-CAFs can originate from resident fibroblasts in response to TGF-β1 secreted by tumors and stroma. Our results open the possibility that targeting TGF-β1-mediated paracrine networks might be a better strategy for sarcoma therapy. In summary, we have demonstrated the role of TGF-β1 on the transition of GFs to CAFs, which describes a detailed mechanism of how CAFs are derived from normal resistant fibroblasts in the sarcoma microenvironment.

## Materials and methods

### Human GIST samples

After obtaining written informed consent, GIST samples were collected from patients undergoing resections at the University of California, San Diego (UCSD). All procedures were approved by the UCSD Institutional Review Board (IRB; #181755). Experienced pathologists made the pathological diagnosis based on light microscopic analysis of FFPE tissue sections labeled with antibodies against KIT (Santa Cruz; sc-13508) and DOG-1 (Abcam; ab190721). Excess tumor tissue was used for only research purposes.

### Cell lines

The human GIST cell line GIST-T1 (*KIT* exon 11 mutation) was from T. Taguchi (Kochi Medical School, Japan). GIST430 (*KIT* exon 11, 13 mutations) and GIST882 (*KIT* exon 13 mutation) were provided by Dr. Jonathan A. Fletcher (Dana–Farber Cancer Center, Boston, MA). Normal GFs were purchased from Science Research Laboratories (Carlsbad, CA), and CAFs were isolated from a human *PDGFRA* mutant GIST by a serial trypsinization method^33^. CAFscr, CAFshPDGFC #1, and CAFshPDGFC #2 cell lines were established using lentiviral transduction^12^. DNA authentication of CAFs was performed by short tandem repeat profiling (DDC Medical, Inc). GIST-T1, GFs, and CAFs were maintained in Dulbecco’s Modified Eagle Medium (DMEM; Gibco, Grand Island, NY) with 10% fetal bovine serum (FBS) and 1% penicillin–streptomycin (Mediatech, Manassas, VA). GIST882 was cultured in Roswell Park Memorial Institute (RPMI 1640; Gibco) with 20% FBS and 1% penicillin–streptomycin. GIST430 was grown in Iscove’s Modified Dulbecco’s Medium (IMDM; Gibco) with 15% FBS, 2 mM glutamine, and 1% penicillin–streptomycin. All cell lines were incubated at 37 °C in an incubator with 5% CO_2_. To monitor *Mycoplasma* contamination, all cell lines were regularly tested using PCR reaction with primers; forward, 5′-GGCGAATGGGTGAGTAACACG; reverse, 5′-CGGATAACGCTTGCGACCTAT.

### Quantitative real-time PCR (qPCR)

RNeasy Mini Kit (Qiagen, Germantown, MD) was used to extract total RNA from GIST lines, CAFs, and GFs. cDNA was synthesized from total RNA (1 µg) by First Strand cDNA Synthesis Kit (Invitrogen, Waltham, MA) according to the manufacturer’s instructions. qPCR was performed using SYBR green (Bio-Rad, Hercules, CA) on the CFX96 cycler (Bio-Rad). The fold changes were normalized using *GAPDH* levels, and each reaction was conducted in triplicate. The primer sequences were as follows: *PDGFC* (forward, 5′-GGCTTCTCCTGCTGACATCT; reverse, 5′-TCCGTTCTGTTCCTTGTTGC); *CCL2* (forward, 5′-GCAGCAAGTGTCCCAAAGAA; reverse, 5′-TCGGAGTTTGGGTTTGCTTG); *RAB3B* (forward, 5′-AACTGCAGATCTGGGACACA; reverse, 5′-ACACTTGTTCCCCACCAGAA); *TNC* (forward, 5’-GAACCTGGTGTCTTCCCTGA; reverse, 5′-AGACACAGCCACATCCTTCA); *TGFB1* (forward, 5′-TACAGCAACAATTCCTGGCG; reverse, 5′-AAGCCCTCAATTTCCCCTCC); *FGF1* (forward, 5′-CTGGAAAGGCTGGAGGAGAA; reverse, 5′-CGTTTGCAGCTCCCATTCTT); *FGF5* (forward, 5′-AGTGGTATGTGGCCCTGAAT; reverse, 5′-CTGCTCCGACTGCTTGAATC); *FGF9* (forward, 5′-ATAAGCACGTGGACACTGGA; reverse, 5′-TGTGTAATTTCTGGTGCCG); *GAPDH* (forward, 5′-TCGACAGTCAGCCGCATCT; reverse, 5′-TACGACCAAATCCGTTGACTCCGA).

### Human phosphokinase array

Proteome Profiler human phospho-RTK antibody arrays (ARY003; R&D Systems, Minneapolis, MN) were tested in GIST-T1, T1 + CAFscr, T1 + CAFshPDGFC #1, and T1 + CAFshPDGFC #2. According to the manufacturer’s instructions, cell lysates containing a total of 500 μg protein were incubated overnight with an antibody array containing 42 anti-RTK antibodies, and the bound phospho-RTKs were detected with anti-phospho-tyrosine antibody-horseradish peroxidase (HRP). Blots were normalized using the 8 anti-phosphotyrosine antibody control spots per filter using ImageJ software (RRID:SCR_003070).

### Western blot analysis

GIST-T1 cells were treated with CM from CAFscr, CAFshPDGFC #1, or CAFshPDGFC #2 for 24 h. The lysates with RIPA buffer (Cell Signaling Technology, Beverly, MA) were subjected to Western blot with the antibodies (1:1000 dilutions) against p-PDGFRA (Cell Signaling Technology, 4547P), PDGFRA (Cell Signaling Technology, 3164S), p-PDGFRB (ABclonal, Woburn, MA, Apo815), PDGFRB (Cell Signaling Technology, 3169 S), and β-actin (Cell Signaling Technology, 4967S). After SDS–polyacrylamide gel electrophoresis (Invitrogen) with the lysates, the gel was transferred to polyvinylidene difluoride membrane (Bio-Rad) with Trans-Blot Turbo transfer system 690BR (Bio-Rad). The immune-reactive bands with the secondary HRP-conjugated antibodies were visualized using a chemiluminescent substrate (Invitrogen) and were exposed to X-ray film (Genesee Scientific, San Diego, CA).

### IHC and H&E staining

Resected human GIST tissues were frozen-embedded in OCT compound and sectioned at 5-µm thickness. Following fixation with 4% formaldehyde, the slides were stained for TGF-β1. Mouse liver tissues collected from the spleen-to-liver metastasis model were formalin-fixed, paraffin-embedded, and sectioned at 5-µm thickness. IHC staining was performed using ABC Universal PLUS Kit (Vector Laboratories, Burlingame, CA). For antigen retrieval, the slides were boiled with IHC Antigen Retrieval Solution (Invitrogen) for 30 min. After eliminating endogenous peroxidase activity with 0.3% hydrogen peroxide, anti-TGF-β1 (Abcam; ab92486) and anti-KIT (Dako; A4502) antibodies were incubated overnight at 4 °C (1:250 dilution). The following day, the sections were developed with HRP-conjugated secondary antibody and DAB chromogen provided with the kit (Vector Laboratories). For H&E staining, the mouse liver slides were stained in hematoxylin solution (EMD Millipore) for 2 min. After washing in running tap water for 5 min, the slides were counterstained in eosin solution (Sigma-Aldrich) for 30 s. The slides of IHC and H&E were photographed using CKX53 microscopy (Olympus).

### IF staining

IF staining was performed using resected tissues from two GIST patients. Briefly, the tumor sections were fixed with 4% paraformaldehyde for 10 min. After washing the slides with PBS, the samples were blocked with 5% goat serum for 1 h at room temperature. The slides were then incubated with anti-c-KIT (Santa Cruz; sc-13508) and anti-TGF-β1 antibodies (Abcam; ab92486) overnight at 4 °C (1:1000 dilutions). The signal was detected with anti-mouse Alexa Fluor 594 and anti-rabbit Alexa Fluor 488 (Invitrogen). Fluorescence images were visualized using a Confocal Microscope A1R (Nikon Inc.).

### Enzyme-linked immunosorbent assay (ELISA)

The levels of TGF-β1 secretion were evaluated using the TGFB1 ELISA Kit (MyBioSource, San Diego, CA). For sample preparation, cell culture supernatants were collected from GF, CAF, GIST-T1, GIST882, and GIST430 cells. After the supernatants were activated by activating reagent (0.2 N HCl) for 10 min, the samples were added into a 96-well microplate coated with TGF-β1 capture antibodies. The captured TGF-β1 was developed by Avidin-Biotin-Peroxidase complex, and the absorbance was read at 450 nm with a microplate reader (BioTek).

### Migration assay

GFs (5 × 10^4^ cells) were seeded with TGF-β1 (InvivoGen, San Diego, CA), anti-TGF-β1 blocking antibody (BioLegend, Cat# 521707), GIST CM or CAF CM in the bottom of 24-well Transwell plates. After we washed out the media with fresh serum-free media, 5 × 10^4^ GIST-T1 or GIST882 cells were seeded in the upper chamber (SARSTEDT Inc, Sparks, NV). The migrated cells adherent to the membrane under-surface were photographed using a BZ-X800 Life Science Microscope (KEYENCE, Itasca, IL). The cells were counted and analyzed by ImageJ software.

### Spleen-to-liver metastasis model

The mouse experiments were conducted and approved in accordance with the Animal Care Committee of the University of UCSD (S11020). Five-week-old male Balb/c nude mice (NU/J-002019) were obtained from The Jackson Laboratory (JAX). After anesthesia, the mice were made ~1 cm incision in the left abdominal flank. GIST-T1 (GFP-conjugated; 5 × 10^6^ cells), GIST-T1 (5 × 10^6^ cells) with GF (1 × 10^6^ cells), and GIST-T1 with GF pretreated with TGF-β1 for 24 h were injected into the spleen. After 3 weeks, livers collected from each group were analyzed using the IVIS imaging system, and the GFP signals produced from livers were graphed by total photon flux (p/s).

### Statistical analyses

Data are presented as the mean ± SEM. Statistical comparisons between two groups were performed with the one-way ANOVA, followed by the Student’s *t* test. Sidak’s multiple comparison test was used to compare among more than two groups. A value of *P* < 0.05 was considered significant.

## Supplementary information

Supplementary Figures and Legends
