# Supplementary material for: TGF-β1-mediated transition of resident fibroblasts to cancer-associated fibroblasts promotes cancer metastasis in gastrointestinal stromal tumor
Source: Oncogenesis. 2021 Feb 6;10(2):13. doi: 10.1038/s41389-021-00302-5 (PMC7876107; doi:10.1038/s41389-021-00302-5)
Supplement: Supplementary file 1 — Supplementary Figures and Legends [file 41389_2021_302_MOESM1_ESM.pdf]

SUPPLEMENTARY DATA

TGF-β1-mediated transition of resident fibroblasts to cancer-associated fibroblasts promotes cancer metastasis in gastrointestinal stromal tumor

***Running Title:** TGF-β1 promotes a CAF transition in GIST

Hyunho Yoon[1,2], Chih-Min Tang[1,2], Sudeep Banerjee[1,2,3], Antonio L. Delgado[1,2], Mayra Yebra[1,2], Jacob Davis[1,2], and Jason K. Sicklick[1,2]*

[1] Department of Surgery, Division of Surgical Oncology, University of California, San Diego, CA, USA
[2] Moores Cancer Center, University of California, San Diego, CA, USA
[3] Department of Surgery, University of California, Los Angeles, CA, USA

*Correspondence: Dr. Jason K. Sicklick, Division of Surgical Oncology, Department of Surgery, Moores Cancer Center, University of California, San Diego, 3855 Health Sciences Drive, Mail Code 0987, La Jolla, CA, 92093-0987, USA. Email: jsicklick@health.ucsd.edu.

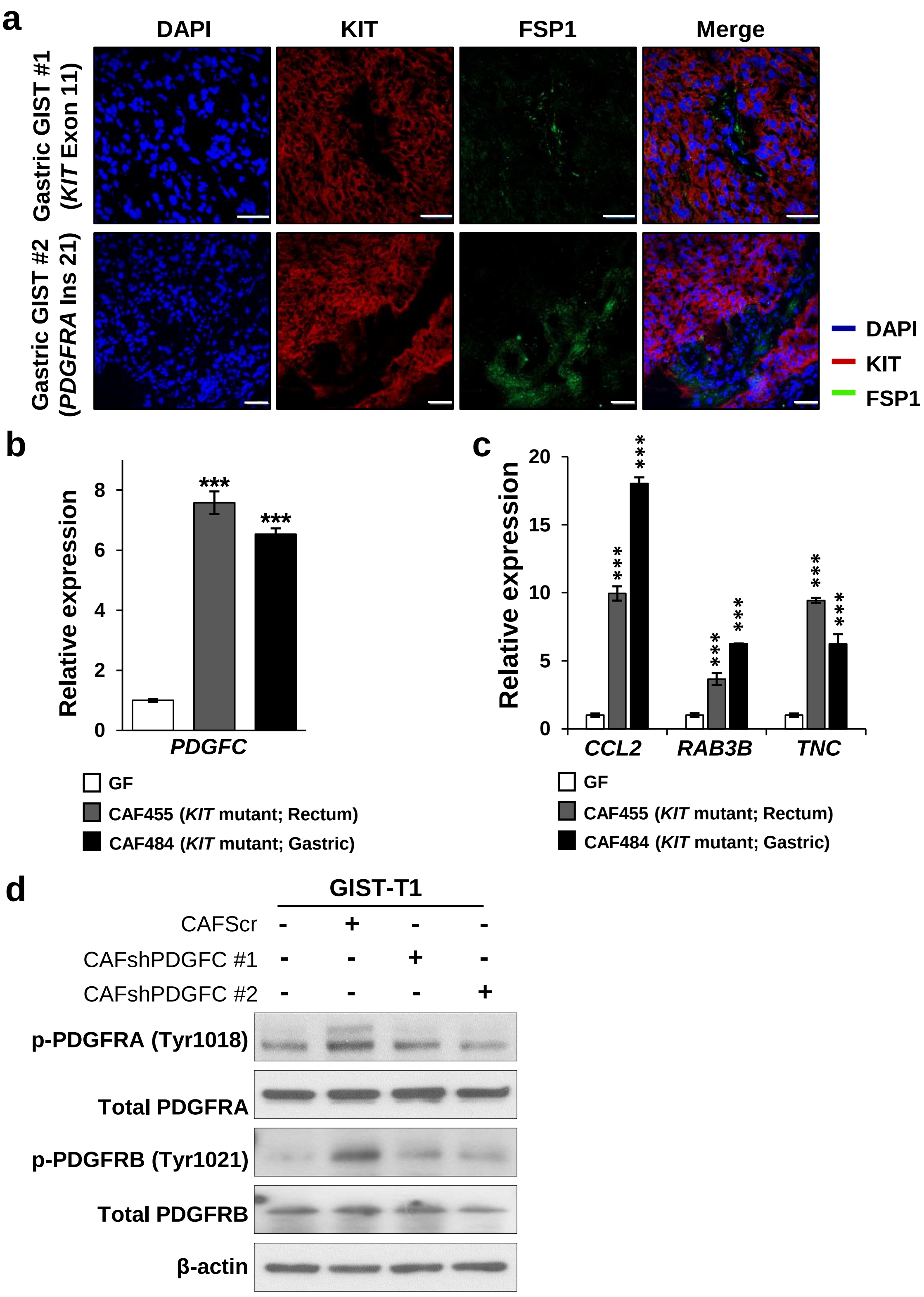

Supplementary Fig. S1. PDGFC secreted from CAFs activates PDGFRA in GIST-T1 cells. a, Immunofluorescence (IF) images of FSP1 (green), KIT (red), and DAPI (blue) staining in the resected sections. Scale bars, 50 μm. **b**, **c**, Expression of *PDGFC, CCL2, RBA3B,* and *TNC* mRNA in GF and CAF lines by qPCR. **d**, The lysates from GIST-T1 with conditioned media (CM) from CAFscr, CAFshPDGFC #1, or CAFshPDGFC #2 for 24 h were subjected to Western blot with the antibodies against phosphor-PDGFRA (p-PDGFRA), PDGFRA, p-PDGFRB, PDGFRB, and β-actin as a loading control.

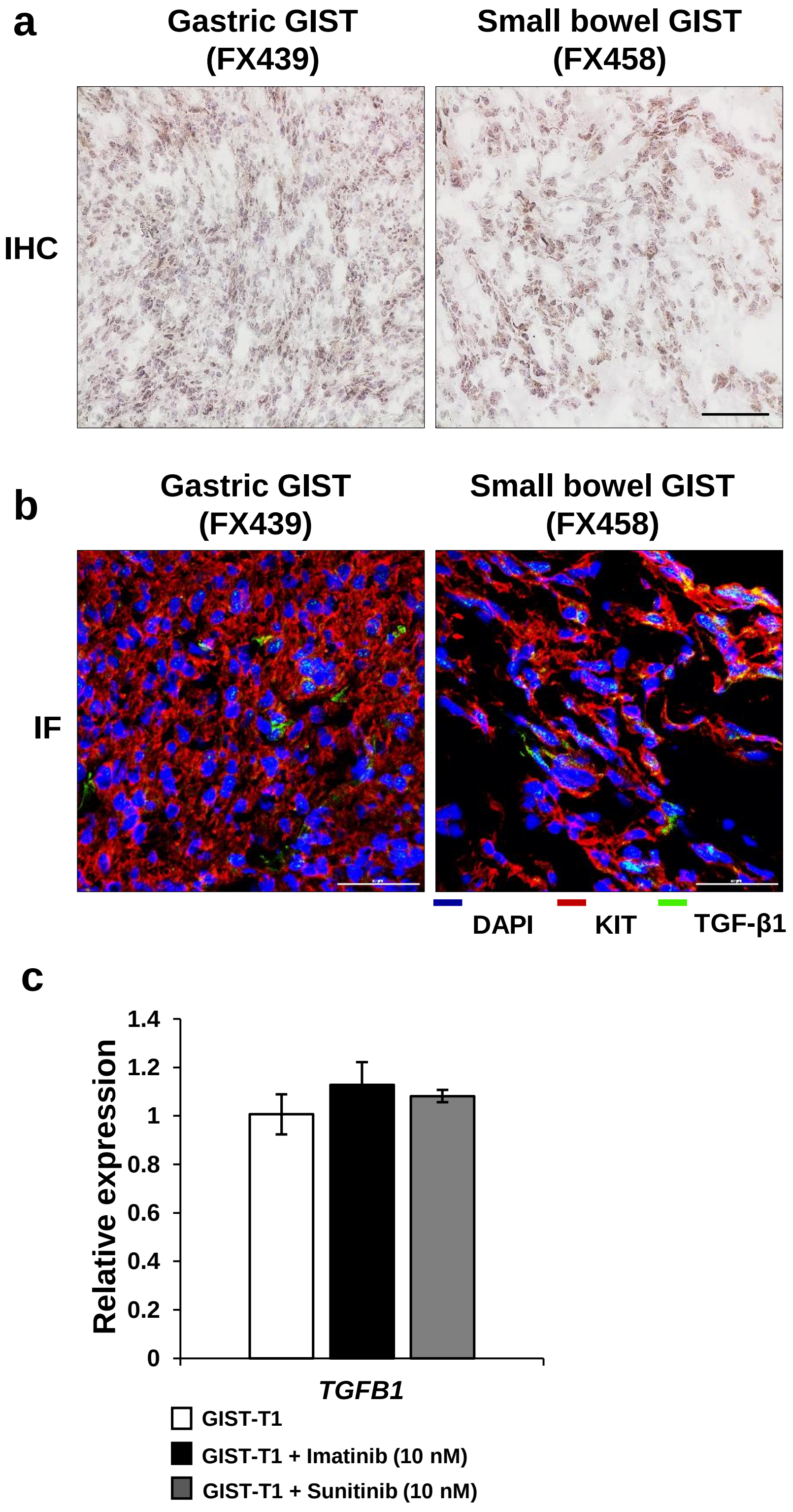

Supplementary Fig. S2. TGF-β1 is highly expressed in human resected GISTs. a, Representative immunohistochemistry (IHC) images with staining for TGF-β1 in the frozen tumor sections collected from human gastric and small bowel GISTs harboring mutant *KIT*. Scale bars, 100 µm. **b**, Representative IF images of TGF-β1 (green), KIT (red), and DAPI (blue) staining in the resected sections. Scale bars, 50 µm. FX439: Gastric GIST, mutant *KIT* exon 11; FX458: Small bowel GIST, mutant *KIT* exon 11. **c**, qPCR mRNA expression of *TGFB1* in GIST-T1 treated with TKIs, imatinib and sunitinib.

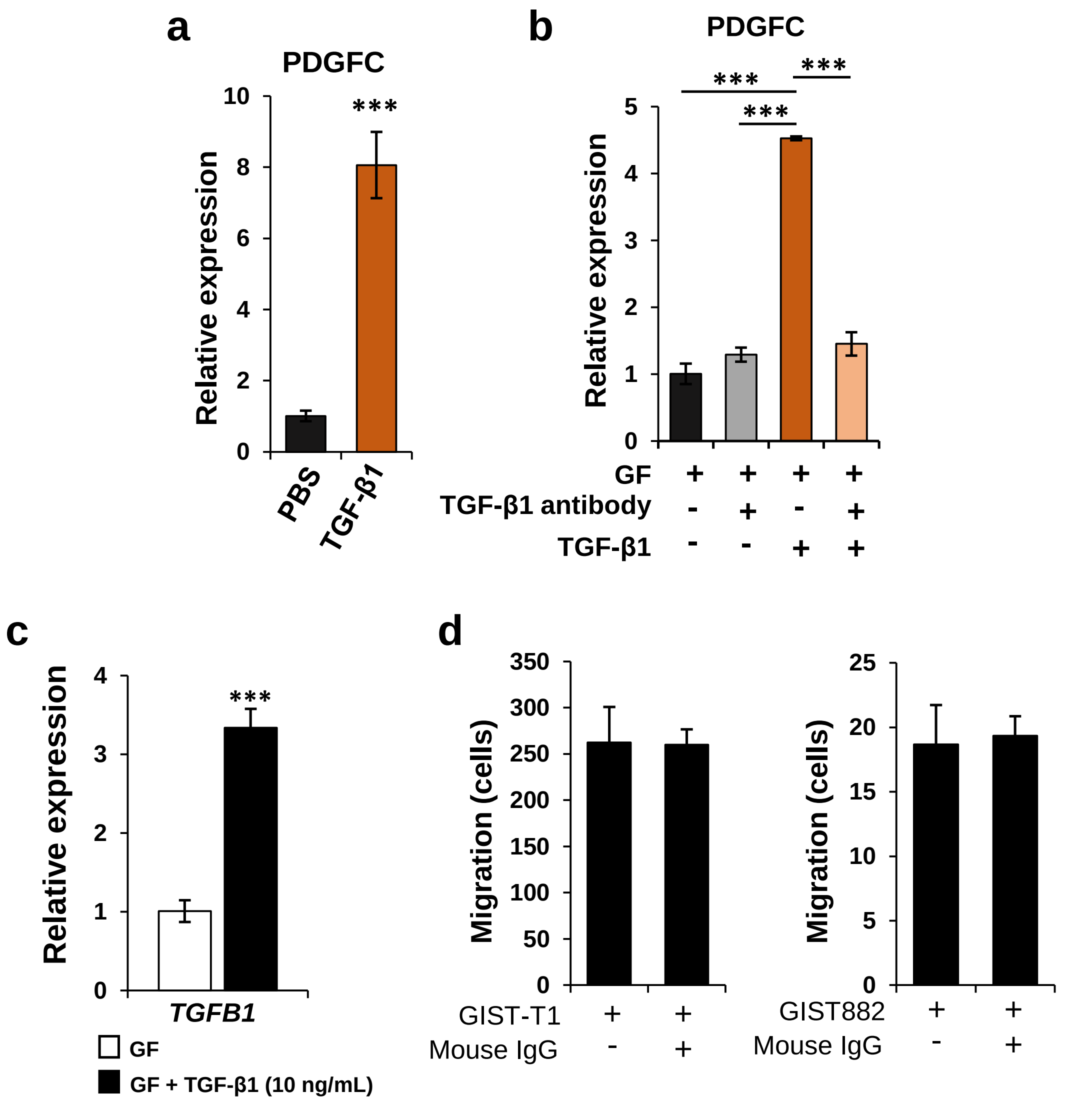

Supplementary Fig. S3. TGF-β1 is associated with a transition from normal gastric fibroblast (GF) to CAF. a, Effect of TGF-β1 treatment on mRNA expression of *PDGFC* in GFs. GFs were treated with TGF-β1 (10 ng/mL) for 48 h. The expression levels of these genes were measured by qPCR. **b**, Relative expression of *PDGFC* in GFs treated with TGF-β1 (10 ng/mL) and/or anti-TGF-β1 blocking antibody (1 µg/mL) for 48 h. All graphs show mean ± SEM, and p-values were represented by Student's T-test or ANOVA analysis. ***p < 0.001. **c**, *TGFB1* mRNA expression in GFs treated with TGF-β1 (10 ng/mL) by qPCR. p-values were represented by Student's T-test. ***p < 0.001. **d**, Effect of an isotype control (mouse IgG) on Transwell migration in GIST-T1 and GIST882.

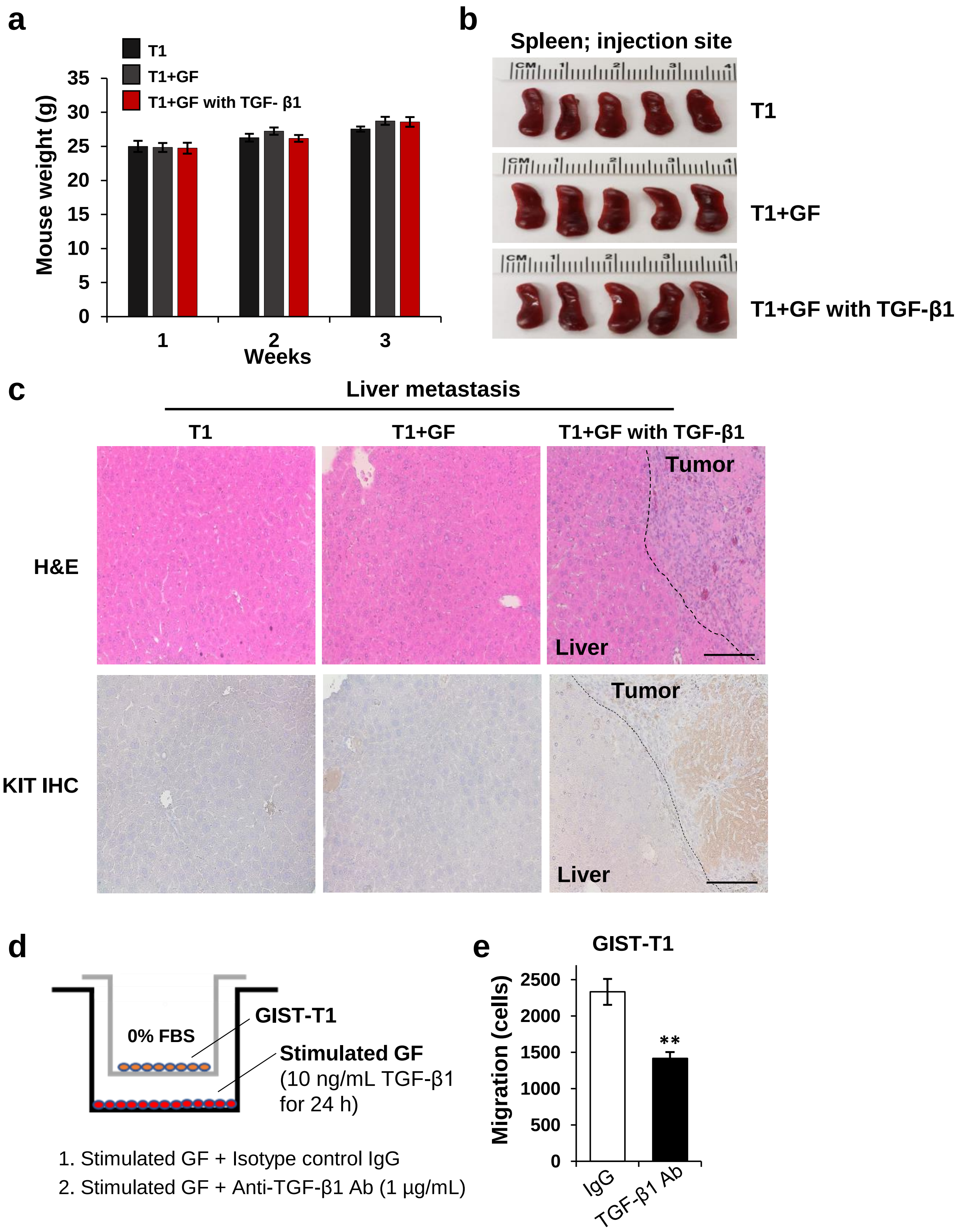

Supplementary Fig. S4. TGF-β1-mediated transition from GF to CAF promotes GIST metastasis in the spleen-to-liver mouse model. a, The mouse weight in each group was monitored weekly after the cells were injected. The graphs show mean ± SEM. **b**, All photographic images of spleen in Figure 6a. **c**, Representative H&E staining images (top) and IHC images (bottom) stained for KIT in the tumor section collected from metastatic livers. Scale bars, 100 μm. **d**, Experimental design for Transwell migration assay of anti-TGF-β1 blocking antibody in the stimulated GF. **e**, Quantitative data with the migrated GIST-T1 cells. *p*-values were represented by Student's T-test, $^{**}p < 0.01$.
